# An Essay on Professional Empiricism

**Published:** 1851-10

**Authors:** A. C. Hawes


### ARTICLE IV.

*An Essay on Professional Empiricism. Read before the American Society of Dental Surgeons, at their Twelfth Annual Meeting, held in Philadelphia, August 5th,* 1851. By A. C. HAWES, D. D. S.

CONNECTED with almost every kind of honest business with which I am acquainted, are to be found some improper and dishonest practices, which arise too often from the minds of money-grasping, vicious men, engaged therein, whose aim is neither the advancement of science or art, nor the elevation of the calling which they have chosen to pursue; but rather the accumulation of the profits arising therefrom, however they may be obtained.

These practices, as applied to distinct occupation have been called—so long that "the memory of man runneth not to the contrary"—"the tricks of the trade." But, in these latter days, they are more properly defined by the general term "*Humbug.*"

I have been invited to prepare a short "Essay" upon some subject connected with the "theory or practice of dentistry," and, I trust, no member of this association will be offended that I have thus early in my effort, arrived at the word "humbug." I hope I shall be pardoned, but I really do not know any subject more positively connected with the "*practice*" of our profession (and I regret to say, sometimes, more profitably) than this modern word, which contains so much disgraceful meaning. Of the "*theory*" I do not design so particularly to speak.

It is said to have been a maxim once taught by a dishonest father to his son—"Get *money!* get it *honestly*, if you can, but get it." And we now and then find a member of our dental faculty, whom to judge from his professional conduct, we might safely suppose to be the veritable son who had taken his father's maxim as law, and felt bound to abide by it.

We witness these professional impositions in the reprehensible want of candor exhibited towards those who patronise such dentists as I have alluded to, in the inclination to deceive them in regard to the condition of their teeth; and the nature of the operation which may so particularly effect their comfort for all coming time. In the lack of sympathy shown to the patient, and the willingness of the operator to forget his comfort; as well as in the absolute recklessness which he manifests for the character of his profession; for, in his eager grasp for the "almighty dollar," he loses sight entirely of the fact, that something is due to the profession and its character. He seems to forget, in view of this corrupting talisman, that he is dealing with flesh and blood, and that the delicate organs which he operates upon, contain nerves and blood-vessels, which render them so extremely sensitive as to require all the skill and care which can possibly be bestowed to prevent the most excruciating pain.

He considers the whole operation a mere business transaction, and comforts himself by thinking that there is no money to be made by entertaining sympathy for the suffering patient.

By such practitioners, the patients are received with an air of kindness, which is, of course, pleasing to them, and while suffering, perhaps, under the pain of a raging tooth, or their minds are filled with a fearful anxiety in relation to the amount of suffering they are to endure in the process of the operation, they are prepared to believe any thing which the dentist may tell them, however absurd. And the frank and open, only in his manner, is prepared to tell them any thing which will tend to swell his profits, though the patient suffer under or by the operation ever so much. If a question is asked, the answer of the dentist is such as would be likely to secure the job ; his object being not to tell the truth—not the establishment of a character—not the elevation of his professsion, but to "get money."

If, for instance, a lady, under a mistaken idea of the manner of performing an operation, and ignorant of the technicalities of the profession, ask him if he will insert a single tooth on the "principle of atmospheric pressure," he readily replies—"Oh, certainly, if you wish it ; that is a very common way for *good* dentists to insert single teeth. It is a little more expensive, to be sure ; but when you want a single tooth *well* set, it should always be on the principle of "atmospheric pressure." There are very few dentists who *can* insert them upon that principle—though, *I* have had much practice in that way ; and you will find it better, by all means, when you have teeth inserted, to have them fastened in that manner." When the operation is completed, he informs his fair "patient," that he has added a little more expense, by fastening a couple of clasps upon each end of the plate, just to *steady* the tooth.

It then being *secured* in the lady's mouth, he takes his pay, for the single tooth, the "atmospheric pressure," and the "four clasps," with a manner of exceeding honesty, and as a parting favor he gives her the gratuitous information, that when the *air* is exhausted, she will perceive that the tooth adheres to the gum most delightfully, and adds, as a parting word, "but, madam, if any thing should happen to *that* tooth, be sure and consult *me*." The lady retires well pleased that she has been through the "atmospheric pressure" operation—with four springs on the plate, with so little pain, and she is delighted with the seeming candor, urbanity and honest bearing of the operator, and is well satisfied to have paid the triple price of his impositions, and the man smiles at his own impudence.

It is true, that such a man cannot long impose on the same person, in this more than any other calling in life; for the time comes when the patient learns, that there is more "*suction*" in the "atmospheric pressure" principle of such a dentist, than is at first bargained for. He is, in short, one of those whom Shakspeare has described as "one who will steal into a man's favor, and for a week escape a great deal of discovery, but when you find him out you have him ever after."

Dr. Berry, of Newport, once informed me that a lady one day called on him, who had had a tooth inserted on the root of another, and desired him to insert a second in the same manner, asserting that it had been in for nearly a year and that she had never had the slightest trouble with it, for the reason that it was fastened by suction.

The doctor examined the tooth, (perhaps rather more minutely than prudence would have dictated,) for the purpose of ascertaining the modus operandi of his professional predecessor; but while prying into (what, for aught we know was) the air chamber, the tooth came out, when lo! the discovery was made, that the suction lay in a large *iron* pivot, which had become nearly rusted off.

Such practices as these I have reason to think are resorted to by many who have adopted our profession, but such a course I know, must be condemned by the great body of our dentists. Those who are really skilful, have no need to resort to deceptions like these, and those who are unskilful, will much sooner arrive at a respectable standing among their brethren, by seeking to improve. If the gain of gold is the only object for malpractice with these wicked cheats, there is nothing which will so surely keep one from the goal of his desires. It certainly cannot be wrong to deal honestly, and it is something more than dishonest, it savors much of cruelty to deceive those who, not only confide in our word, and who, suffering with agony, in all confidence place themselves in our hands, to endure a still greater amount of real or imaginary torture. When or where do we see more intense agony than we observe depicted in the countenance of a timid person when about to submit to some dental operation, however simple. When anticipated pain like an electric shock, seems to dart through every fibre of the delicate frame, and "cold fearful drops stand on the trembling flesh."

I have seen patients laboring under such a weight of painful excitement, when only awaiting the extraction of a tooth, that if instead of a tooth, life itself were about to be parted with, they scarce could suffer more. And surely this is no time for deception—this is not the occasion on which to practice the "tricks of trade." This is the moment in which the dentist is looked to with the utmost confidence, and this is when he is called upon to exhibit, not only truthfulness, but sympathy, to speak a word of encouragement, to allay the (perhaps needless yet still oppressive) fears. And to my mind it is as much a part of the duty of a dentist to prepare the *mind* of the patient for an operation, as it is to perform that operation in a faithful manner.

The dental art is but in its infancy. Each succeeding year brings to light new discoveries and important improvements. And the experience of every day shows us that the business of the skilful dentist is more and more appreciated. Then away with all deceptions, all tricks, all "humbugs." Let industry and honest emulation among our members be the means to excellence; let our aim be, not so much to get rich, as to raise a standard of honorable character for our profession, and for ourselves. Industry, integrity and a constant aim at perfection in our art, will bring to us wealth and character, the praise of men, and the approving smile of heaven.

